# The 3D Value Framework for Sustainable Access to Effective Antibiotics

**DOI:** 10.1007/s10728-025-00552-z

**Published:** 2025-12-06

**Authors:** Anders Herlitz, Erik Malmqvist, Christian Munthe

**Affiliations:** 1 Department of philosophy, Lund University, Lund, Sweden; 2 Institute for Futures Studies, Stockholm, Sweden; 3 Philosophy, linguistics and theory of science, University of Gothenburg, Gothenburg, Sweden; 4 Centre for antibiotic resistance research in Gothenburg (CARe), University of Gothenburg, Gothenburg, Sweden

**Keywords:** Antibiotics, Antimicrobial resistance, Cost-effectiveness analysis, Healthcare access, Health policy, Sustainability, Value

## Abstract

Sustainable access to effective antibiotics is a main rationale for addressing antimicrobial resistance, for calls to enhance antibiotic supply security and for development support to promote health system capacity in low- and middle-income settings. Standard models for valuing pharmaceuticals fail to capture the full value of sustainable access to effective antibiotics. In effect, assessments of societal or corporate investments to promote such access underestimate their value and the cost of foregoing them. In effect, measures that could address existing sustainability problems in antibiotic supply systems are hampered. We review the features needed to avoid these problems and assess suggestions to address the flaws of existing approaches, such as the STEDI-model. On this basis, we propose the *3D Value Framework*, a straightforward and comprehensive model that focusses directly on *what types of valuable outcomes there can be* related to having sustainable access to effective antibiotics. We also sketch how this model can be used in cost-effectiveness analysis grounding public decision-making and discuss further applications in other settings.

## Background

Sustainable access to effective antibiotics is of paramount importance for health systems, local and global public health [1–4]. At the same time, such access is undermined by several factors. Weak and vulnerable supply chains negatively impact all health systems [5–7]. Antibiotic resistance (ABR) due to unsustainable consumption [8, 9], environmental pollution [10–12] and slow antibiotic innovation [13–15] undermines therapeutic effectiveness. A recent analysis finds ABR to be associated with 4.7 million global deaths in 2021 alone, projected to rise to 8.2 million in 2050 [16]. To this can be added heavy burdens, remaining mental and physical scars and dysfunction for those who survive [17]. Finally, lack of economic capacity in low- and middle-income (LMI) settings broadly prevent access even to effective products otherwise available on the market [1, 18, 19]. Already in 2020, this factor was estimated to cause 5.7 million deaths globally [20]. The argument in this article is relevant to all these threats to sustainable access to effective antibiotics in LMI as well as high-income (HI) settings.

Access can thus be hampered by weak supply security and/or lack of means to buy what is available. Effectiveness can be undermined by both these factors, plus ABR. Lack of sustainability, finally, is about how all such factors – alone or in combination – create a negative overall dynamic increasingly diminishing access to effective antibiotics over time [21]. Sustainable access to effective antibiotics thus requires health systems and people in need to have real and similar access to effective antibiotics globally and across time [22, 23].

To counter threats undermining access, therapeutic effectiveness and sustainability, societies need to invest in measures that promote sustainable access to effective antibiotics [1, 18, 22]. To achieve this, decision makers must determine how to purchase and/or subsidize antibiotics, incentivize research and development of new antibiotics, create robust arrangements to promote local and global antibiotic supply and access to antibiotics, and mitigate ABR to preserve antibiotic effectiveness. These areas need to be addressed in a balanced fashion to avoid counterproductivity or ineffectiveness due to the complexity of the challenge. For instance, actions to reduce antibiotic use or to incentivize industry to limit emissions to mitigate ABR may cause reduced antibiotic innovation, elevated prices or even market abandonment, all of which undermine sustainable access to effective antibiotics [7, 18, 24, 25]. To counter such threats, governments and health systems may ponder so-called “pull-incentives” to reward industry actors economically for innovation and sustainable supply [26], or subsidies and capacity-building development support to make antibiotics better available – or less needed, by reducing infection burdens – for economically disadvantaged groups and regions [1].

Each of these types of intervention presents a multitude of possible types of actions, all of which potentially generate substantial societal costs – direct monetary costs as well as indirect running costs and opportunity costs of foregone action in other areas [27, 28]. This creates a threat of ever-increasing spending and intolerable opportunity costs, necessitating limits for how large costs may be accepted [21, 28, 29]. These costs, therefore, need to be balanced against expected benefits of increased sustainable access to effective antibiotics in a cost-effectiveness analytic fashion. But to adequately assess these benefits, it is crucial to appropriately conceptualize the values of sustainable access to effective antibiotics – i.e., what types of good may come out of it (and be lost when the access is lacking). Otherwise, there will be no basis for evaluating whether the costs of a proposed measure to promote sustainable access to effective antibiotics can be justified.

In this article, we demonstrate that even the most advanced existing theoretical models for valuing (different aspects of) sustainable access to effective antibiotics in the context of cost-effectiveness analyses have serious limitations. We therefore present the *3D Value Framework*, a new taxonomy designed to more comprehensively capture the full value of sustainable access to effective antibiotics across three main dimensions.

After positioning this proposal methodologically and theoretically, we first highlight the special role of antibiotics in health systems and how it grounds several sources of the value of sustainable access to effective antibiotics. This analysis motivates a set of general requirements that any satisfactory evaluation model for sustainable access to effective antibiotics must meet. We then review the shortcomings of the standard approach to valuing pharmaceuticals in relation to valuing antibiotics, as well as some recently presented extensions of this approach aimed at overcoming its downsides. On this basis, we present the 3D Value Framework and discuss how it may be used to improve cost-effectiveness analyses grounding policy- and decision-making to address sustainable access to effective antibiotics in different areas, as well as on different levels in health systems.

## Method and Approach

Like the standard approach to valuing pharmaceuticals, and recent extensions of it reviewed below, the 3D Value Framework addresses an evaluative and normative challenge occurring somewhere in between basic ethical and economic theory and the practical application of cost-effectiveness analysis. With respect to ethics, it does not assume any particular comprehensive ethical theory (e.g. consequentialism or contractualism) but rather relies on assumptions (made explicit and defended at relevant places below) that are consistent with most such theories. In this regard, the 3D Value Framework resembles so-called mid-level ethical principles commonly proposed for healthcare and health policy, such as the well-known and widely accepted four principles of biomedical ethics [30] or widely recognized frameworks for the ethics of public health [31] and health policy [32]. At the same time, it differs from such principles in that it seeks to provide a higher degree of concrete practical guidance.

Similarly, the framework is meant to be consistent with basic economic theory. It is not intended to replace the health economic standard tools described below, but focuses on what information should be fed into existing such tools. Specifically, we assume that societies, health systems and people can form evaluative preferences for the type of factors described by the framework, and that these preferences, together with relevant empirical facts, can govern the ascription of value to be balanced against costs and opportunity costs in economic calculi according to common assumptions in economic theory. We also cite some familiar examples of how such ascription of value are already made in several other areas of policy and life.

More precisely, the 3D Value Framework is supposed to add a broader taxonomy for what types of value may motivate costs when deciding on investments to boost sustainable access to effective antibiotics. As such, it provides input to whatever specific practical tools and procedures for cost-effectiveness analysis and decision-making are already in use in different policy contexts.

Methodologically, we have arrived at our proposal via a theoretical analysis informed by multiple sources, besides the background already described. First, by formulations of the challenge of properly valuing antibiotics recently made in different settings in the literature, and by main attempts presented to meet this challenge (to be described in more detail later). Second, by interactions with stakeholders from health systems engaged in issues about access to antibiotics, resistance management and sustainable pharmacological practices (practitioners, public agency officials, public policy makers, researchers, pharmaceutical providers and developers). This interaction was greatly facilitated by the social innovation platform project PLATINEA, by providing a structure where all of these types of stakeholders and ourselves could interact free of any specific economic dependencies (see: https://www.platinea.se/w/pl/en). Combining these sources have led us to the broad conception of the challenge presented explained at the outset of this article, and to observations of flaws in existing proposals to meet the challenge. These observations are presented and explained under way in the section on standard approaches and recent extensions.

## Sources of Value and Basic Requirements

The results of our analysis are here presented in four stages: First, the analysis of the sources of value of sustainable access to effective antibiotics, grounding five requirements to be met by a satisfactory approach to evaluation. Second, a matching of existing approaches to evaluation of sustainable access to effective antibiotics against the requirements. Third, a presentation of the 3D Value Framework, demonstrating how it meets four of the requirements. Fourth, a demonstration that the 3D Value Framework meets the fifth requirement of practicality.

Antibiotic products stand out compared to most other health-related resources, including pharmaceuticals, in two crucial ways. First, the use of antibiotics generates *more types of value* than most other resources. Second, *continuous access* to antibiotics is fundamental to the overall functioning of a health system in a way that access to other pharmaceuticals rarely is. This observation has led many to suggest that the way in which access to effective antibiotics is evaluated, both related to resistance management, to antibiotic innovation and to development support to decrease needs of antibiotic or economic access, needs to be reformed [18, 28, 33–40]. We will here, first, work through these sources of value systematically before moving to the question of fitting requirements of a valuation framework for sustainable access to effective antibiotics. Important aspects of specific contributions – upsides, flaws as well as differences – will be considered in the next section.

Health-related resources, such as pharmaceuticals, typically generate their primary value through the immediate therapeutic benefit they can provide to people with health needs. This is recognized as something valuable in all frameworks of healthcare ethics and health policy. An appendectomy is valuable primarily in virtue of the health benefits a patient with an inflamed appendix attains from having their appendix removed. A heart transplant is valuable in virtue of the health benefits a patient with a failing heart attains from receiving a new organ. The value of metformin is derived from the health benefits a person with diabetes gains from decreasing the amount of glucose in the blood. Antibiotics do have this same type of value but also generate a range of other values in terms of met individual health needs.

The value of antibiotics is *partially* derived from direct health benefits to patients with bacterial disease (e.g., the benefits of curing a urinary tract infection, strep throat, syphilis, sepsis, pneumonia and so on). Antibiotics are additionally highly valuable to patients because a broad range of medical procedures require access to them to manage infection risk (e.g., surgery, intensive care and many cancer treatments) [41]. Lack of such access makes these procedures much more risky, less effective and possibly infeasible or medically unjustified. Thus, antibiotics can have a secondary or indirect value in terms of individual health needs for a broad range of patients. Moreover, because of these primary and secondary sources of value, access to effective antibiotics also make health systems more cost-effective, so that more health benefits can be generated for more individual patients over a longer time [28, 35, 40].

Taken together, these types of value for individuals also create values of sustainable access to effective antibiotics on a population-level, and through the societal importance of a healthy population [28, 35, 37]. While some of these will be visible as individual health benefits, others will not, albeit systematically generated and captured by standard metrics of population health (such as weighted averages of statistical life-expectancy, morbidity and mortality, distribution of health-related capabilities, and so on). The promotion of population health is generally recognized as the main goal in public health ethical frameworks, often partly motivated by its instrumental importance for other societal goods. Additionally, any mitigation of the resistance challenge will be valuable on this level by prolonging the population level benefits over time. The social importance of a healthier population over a longer time, in turn, includes aspects of a good society beyond health, such as economic prosperity, social stability and available capacity for central societal tasks, e.g., education and national defence.

Both these types of values (patient values and population values) accrue through consumption of antibiotics. However, due to the fundamental role of antibiotics for the function of health systems (effectiveness of many types of treatments, population health and societal goods dependent on that), there is also an important value of the *security* associated with just *knowing* and *being able to count on* that appropriate effective antibiotics are available. While the value of security is not as immediately visible in standard ethics frameworks of relevance, they are generally present in the background and expressed by, for instance, evidential requirements to back up claims about good health effects of procedures, and as assumptions that a certain level of preparedness and readiness to attend to a wide variety of possible health problems is a generally desirable feature of a health system. In basic ethical theory, the notion of this type of values can be grounded in notions about the duty of care, caution and responsibility [42–44]. Whereas certain other health-related resources (notably vaccines) also have value beyond their direct patient benefit and share some of the population-level sources of value with antibiotics, antibiotics are arguably exceptional in linking to such a broad combination of strategic, population-level and security values.

Based on this general analysis of the relevant sources of value, we can now formulate basic requirements that any sound model for valuing sustainable access to effective antibiotics must meet. The first three of these – patient value, population value and security value – concern the types of value that a sound model must be able to capture. The last two – comprehensiveness and practicality – are more about the structural and methodological features of the model. We end this section by setting out and explaining each requirement in turn.

- *Patient value* An evaluation model should capture and reasonably reflect all expected health-benefits for individual patients of consuming (specific) antibiotics.

These benefits include the value of having a bacterial disease successfully treated or prevented, but also the value of prophylactic or preventive treatment to enable other types of treatment (surgery, cancer treatments). In addition, it includes all health benefits for individuals enabled by a more efficient healthcare system that accommodates more patients at a quicker pace.

- *Population value* An evaluation model should capture and reasonably reflect all the expected values to society of antibiotics being consumed which are not visible as patient values.

This value includes all statistical improvements of population health (such as an equity adjusted, weighted average of individual health levels, health opportunities and/or capabilities in a society) which befall any particular individual. It also includes how this population health improvement is extended across time by maintained or increased antibiotic effectiveness. In addition, it includes the social (e.g., economic or political) value of having a healthier population.

- *Security value* An evaluation model should capture and reasonably reflect the value of maintained or increased security of having continued access to effective antibiotics at both the individual and the population levels.

This value includes what it is worth for individuals to have sustainable access to effective antibiotics even if they never in fact consume these antibiotics to generate patient value. It moreover includes what it is worth to society to have sustainable access to effective antibiotics, regardless of the population value realised when antibiotics are in fact consumed.

- *Comprehensiveness* An evaluation model should be able to capture and reasonably reflect all values, whichever they are, that may motivate the costs of investments to maintain or increase sustainable access to effective antibiotics.
- *Practicality* An evaluation model should be feasible to incorporate into or complement existing cost-effectiveness approaches used in health policy decision-making, while remaining simple and straightforward enough to be used by policy makers when deciding on antibiotics-related measures or policies.

A minimal condition for this last requirement to be met is that constituents of the model should be possible to include in or add on to existing health economical frameworks. In addition, it should allow for the use of accessible empirical data to ground decisions based on the peculiarities of specific antibiotic compounds and contextual differences related to supply availability, procurement capacity and ABR.

## State of the Art and Recent Extensions

Although their implementation and exact role in policy making may vary, most health systems today use a standardised type of economic evaluation of pharmaceuticals, implementing the general idea of an effectiveness analysis to compare possible investments to secure access to drugs [45]. The health benefit to a patient who consumes a drug is compared to its costs. The value of health benefits is usually expressed using a measure like Quality-Adjusted Life-Years (QALYs) whereas costs are typically measured in financial terms. The approach aspires to assess how effective a pharmaceutical is to help decision makers determine whether it is worth paying a certain price for it.

Criticism of this standard approach has regarded failure to appropriately balance benefits (however measured) and opportunity costs [46], ethical issues with regard to appropriate ways of capturing the clinical benefit for patients using specific measures such as QALY [47, 48], and challenges to consider long-term dynamic effects and the sustainability of resource allocation decisions [21–23, 49]. As we are here focussing especially on the case of antibiotics, we will not delve deeper into these general debates.

As indicated in the preceding section, it has been recognized for some time that this standard health economic approach is too simple to capture the full value of antibiotics. More complex approaches have therefore been suggested and are also being increasingly tried out in practice [28, 33, 35–40]. Some of these suggestions are rather vague, stressing the importance of the strategic importance of antibiotics and of the population level importance of mitigating the challenge of ABR and sustainable antibiotic access, but others suggest more detailed proposals. A review from 2021 summarizes the more elaborated suggestions (among these, the STEDI approach presented in more detail below), demonstrating that these primarily seek to address the challenge of funding antibiotic innovation, and are typically premised on the idea of identifying predefined causal routes to generate value [37]. The review summarizes seven such routes from the literature and recommend that all of these are combined when valuing increased access to antibiotics, but that all routes, except the immediate value to a patient who is treated for bacterial disease, are only to be viewed as values to society. However, this proposal exhibits several shortcomings. First, since it brings together several suggestions from diverse sources, it involves instances of double counting (e.g., the item of *Novel action value* seems to be already included in *Diversity value* – since the value of a novel antibiotic mechanism lies in its contribution to increased diversity of antibiotic compounds). Moreover, important items reserved for being only of value to society seem also to provide value to individual patients, e.g., the *Enablement value* category, meant to capture the strategic value of antibiotics for other healthcare procedures than treatments of bacterial infection.

Several of these initial problems have been addressed in what has become the most prominent among the suggestions for extending standard health economical approaches to valuing antibiotics, *The STEDI Framework* [38, 39]. Like many other suggestions in this area, STEDI was primarily developed to improve cost-effectiveness analysis related to investments in antibiotic innovation and has recently been applied in the UK by the National Institute for Health and Care Excellence (NICE) [50]. Therefore, presentations of STEDI focus mostly on individual patient benefits. However, nothing seems to preclude extending the main ideas of STED to capture values at the population-level in subsidy decisions for new antibiotics [35]. Moreover, a recent contribution suggests applying STEDI to actions to manage ABR, where values also mainly accrue on a population-level [28]. In addition, it is easy to imagine how further extensions could be made to accommodate measures to promote supply security. In effect, we will assume that STEDI is capable of recognizing patient and population values. Because of these features, STEDI is the most promising attempt to capture the full value of sustainable access to effective antibiotics to date.

STEDI captures several values that antibiotics generate due to their strategic importance. Its core is to distinguish the following causal pathways through which an antibiotic can be valuable in ways going beyond the immediate value for a patient in need of an antibiotic to treat an infection [38]:

- *Spectrum value* The value of using narrow spectrum rather than broad-spectrum antibiotics to treat diseases (for better treatment and to slow down development of antibiotics resistance).
- *Transmission value* The value of preventing transmission of an infectious disease to uninfected individuals.
- *Enablement value* The value arising from the fact that antibiotics enable other medical procedures, such as complex surgeries.
- *Diversity value* The value of diversifying the use of antibiotics to slow down development of antibiotics resistance and boost antibiotic effectiveness.
- *Insurance value* The value of having a reserve of antibiotics in case a catastrophic event occurs.

Some of the constituents of STEDI extend the attention to types of patient value ignored by the standard approach: this regard the *spectrum*,* transmission*,* enablement* and *insurance* values, which may all be applied to this effect. At the same time, STEDI was construed with only one aspect of improving sustainable access to effective antibiotics in mind, namely antibiotic innovation. Extending the model to antibiotic stewardship investments, as proposed by Roope and colleagues [28], widens the scope somewhat but still captures only parts of what may be involved in maintaining or improving sustainable access to effective antibiotics. Both attempts thus limit themselves to model only some means to improve the value of sustainable access to antibiotics, rather than this value itself.

For instance, there are many ways in which antibiotic effectiveness may be boosted besides through the diversity or spectrum values of STEDI [28, 41]. Moreover, protecting antibiotic effectiveness goes beyond optimization of use, and includes investments to reduce the general burden of infection, and to limit environmental emissions that may drive resistance development. In addition, it is unclear how STEDI could capture the full value of investments to improve *economic* access to effective antibiotics in LMI settings, as this value has little to do with either antibiotic innovation or overuse, and will go far beyond health benefits, especially on a population level [18, 51].

However, the most obvious gap in STEDI regards the *security values* of sustainable access to effective antibiotics. As construed in STEDI, the notion of Insurance value is the one supposed to capture this aspect. However, insurance value in STEDI is explicitly meant to capture only the value of preserving a new antibiotic as a last line option in case all other options fail and the value of being able to deal with major catastrophic outbreaks [38]. This does capture one aspect of security, namely the value of having access to effective antibiotics in emergencies. But, as discussed earlier, the full value of sustainable access to effective antibiotics is realised across all patient and population value aspects, and not only in special emergency circumstances but as a matter of daily routine in a health system. The very *threat* of not having access to effective antibiotics significantly limits a health system’s ability to plan and organize its activities. The value of security provided by having sustainable access to effective antibiotics thus remains even if disaster never strikes, as people and societies may count on a robust effective health system, rather than living in a situation of lingering threat to health, wealth and social order. Therefore, it should be recognized that having continuous access to effective antibiotics has substantial value, over and above the value realized when these antibiotics are used.

In this light, the very analogy to insurance solutions (suggested by the term *insurance value* in STEDI) to manage risk and uncertainty as a slogan for security starts to look problematic. The value of an insurance does come from the security it brings. But this security is limited by the special way in which it is produced, namely via promise of retrospective compensation in case of unforeseen harm. The full social value of antibiotic security on a population level, however, far supersedes such considerations, and is not so much about compensation as it is about everyday *preparedness* – e.g. the preparedness brought by the general ability to organize and run a health system knowing that effective antibiotics are continuously available.

## The 3D Value Framework

**Fig. 1:**
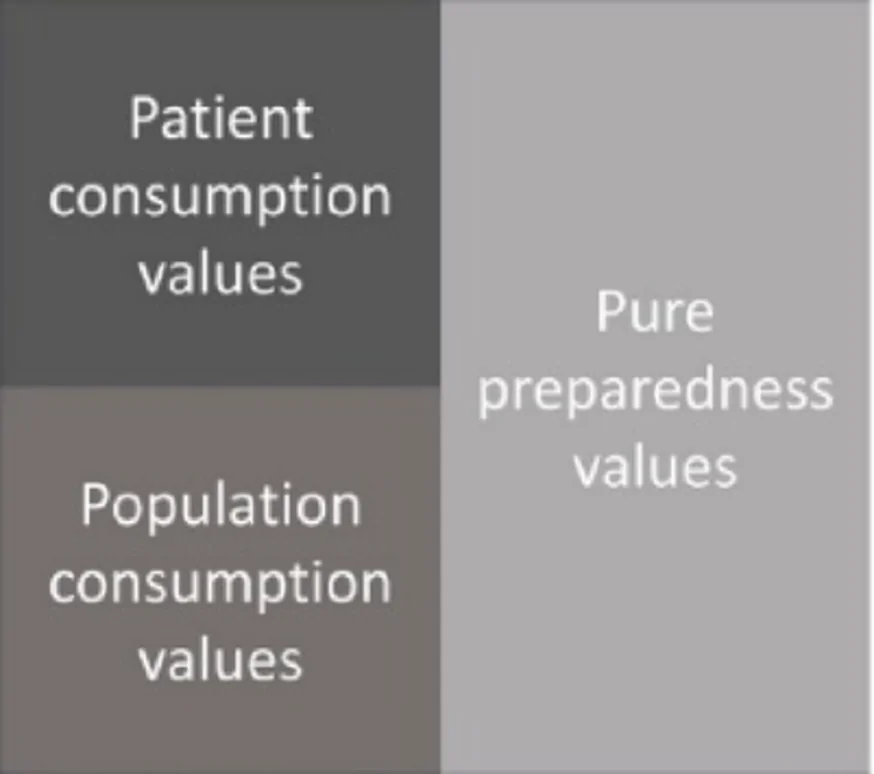
The basic structure of the 3D value framework: its three main dimensions of the value of sustainable access to effective antibiotics

Based on the considerations above, we suggest conceptualizing the types of values that antibiotics generate in three general dimensions, reflecting the differences in value-making features. This approach replaces STEDI's (and similar suggestions’) focus on *causal pathways to uncategorized types of valuable outcomes* with a focus on *what types of valuable outcomes* of sustainable access to effective antibiotics there can be. Using two combined distinctions, we hereby suggest three main dimensions of value. The first two comprise different types of *consumption values*, based on a distinction between (individual) patient values and (collective/societal) population values, as these have been explained earlier. The third dimension comes out of the distinction between consumption values (generated by using effective antibiotics) and what we will call *pure preparedness* values, i.e. the security value of *having access to* effective antibiotics. Since the model does not presuppose any specific causal pathway in which antibiotics may become valuable in these ways, we hypothesize that it has a better chance of approaching the requirement of comprehensiveness than previous suggestions. The resulting basic structure of what we have named the *3D Value Framework* is illustrated in Fig. 1.

The primary patient consumption value of antibiotics is, of course, the health benefit for someone treated for bacterial disease. However, as mentioned, antibiotics also have a secondary benefit for patients by reducing the burden of disease and by improving many other procedures by mitigating infection risks. In a tertiary stage, there is the further strategic value for patients who can access (more or better) treatments of any kind because resources are used more effectively due to the primary and secondary patient values of antibiotics. All these values are instances of *patient consumption value* and may be measured in terms of expected QALY or some other measure of individual health benefits.

Population values differ from patient values in that it is impossible to identify (even in theory) exactly who receives exactly what benefits when the population value is generated. A primary aspect of this is that more of individual benefits will systemically promote statistical population health but not in a way that is visible through specific, identifiable patient values of the same magnitude as this average. Moreover, maintaining or prolonging the effectiveness of antibiotics holds structural importance by sustaining the population health value of sustainable access to effective antibiotics across time. In addition, all population health values have instrumental importance for societal progress and resilience, via its effects on the general economic wealth, social stability and available social capacity. The population values can be measured in terms of aggregated metrics of expected QALY (or other public health measures), combined with standard measures of societal progress and welfare.

As explained, these consumption values all refer to the good coming from *actually using* antibiotics. The pure preparedness values, in contrast, are generated by sustainable access to effective antibiotics *independently of their actual use*. These come out of the security generated by being better prepared for any situation when antibiotics are needed (hence the name). These values can be thought of as an extra dimension added to each type of consumption values. Even if someone will never contract a bacterial disease, there is a security value for this person in knowing that effective antibiotics would be available if they did. And the same holds for any condition calling for healthcare interventions that depend on access to effective antibiotics. The same holds if we consider a person that stays healthy and does not need to take advantage of the increased capacity of a more effective health system; the extra security this person enjoys through this increased effectiveness holds a value in its own right. On a population level, the security brought by better population health over a longer time adds to the value of a more secure society. Besides being more comfortable to live in, it will generally be wealthier, more socially stable and cohesive. Politically, it is common wisdom to value preparedness in the case of (civil and military) national defence for exactly these reasons, even if no war breaks out at the end of the day.

The pure preparedness values are not about concrete health benefits from consumption of antibiotics and may therefore be thought to present a greater challenge to measure. Adding a probability weight to the QALY measure, i.e. a measure of *expected* QALY, may capture a portion of the preparedness value, as it takes risk into account. One way to clarify what such expected QALY would be to assess the *costs* of lack of access to antibiotics [7, 18, 28, 33–35, 37, 52].

However, a major portion of the pure preparedness value cannot be captured in terms of what you would gain if you were to consume an antibiotic weighted by some probability. A main reason to ascribe value to preparedness is that one values precaution and security in its own right, i.e. one holds a risk-aversive attitude. However, expected value measurements (e.g., expected QALY) is neutral with respect to risk (e.g., 0.01 chance of 100 QALY is just as good as 1 QALY for certain), and thus incapable of expressing this kind of value of security for its own sake. To express this precautionary element of preparedness value, one needs to formulate a preference for risk-avoidance by increasing the value one ascribes to creating or maintaining a mere capacity of antibiotic consumption and expected consumption with some fixed-size weight. To decide this weight, societies must decide on how much they value a given level of security (for its own sake). This is something that people do in their private lives when deciding how much money to save for the future, and what all governments do when engaging in economic policy planning or, as pointed out, setting aside capacities and resources for the national defence in peacetime.

**Fig. 2:**
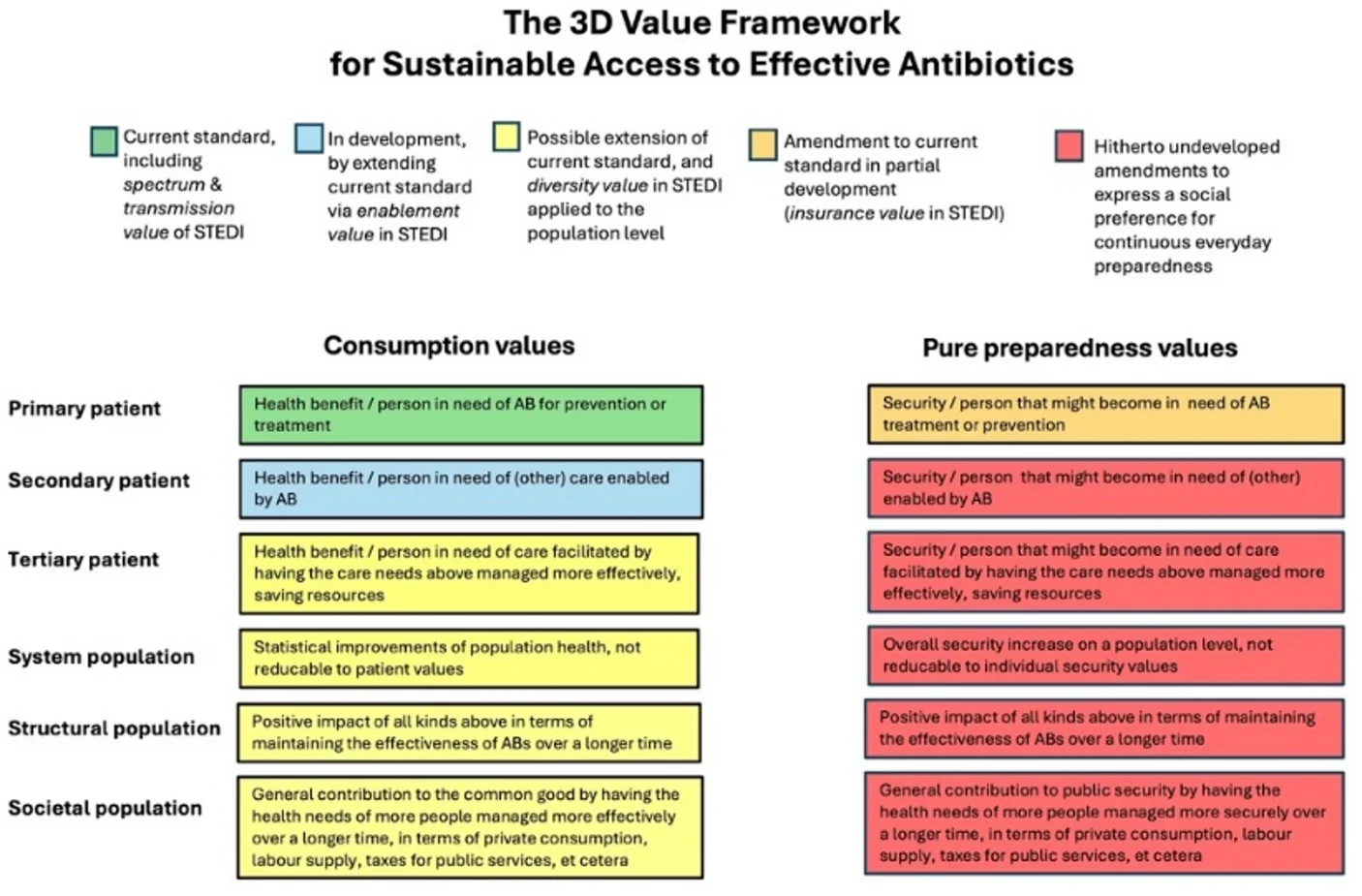
The full 3D value framework model broken down into parts, indicating how much development and/or amendment to current standards and previous extensions they require

Although the 3D Value Framework departs from the STEDI focus on causal pathways to value in favour of types of valuable outcomes, it still incorporates the improvements of STEDI compared to the standard approach to evaluation of pharmaceuticals. Figure 2 provides a more detailed breakdown of the constituents of the 3D Value Framework that have just been outlined, and relate these to the STEDI framework, as well as the degree of development needed to make these constituents operational.

## Applying the 3D Value Framework in Real Decision-Making

The last step is to clarify how the 3D Value Framework may be used in real-world decision making to meet the practicality requirement. The idea is that the evaluation produced by the framework should be fed into a cost-effectiveness analysis that grounds decisions regarding investments to maintain or improve sustainable access to effective antibiotics. We have indicated that both types of consumption values can here be measured using standard methods from health and welfare economics. The secondary and tertiary patient values, as well as the structural and societal population values require additional facts compared to what has typically been considered in standard health economic models for evaluating pharmaceuticals. However, this challenge is shared by STEDI, where it is recognized that the transmission and enablement values of antibiotics (in terms of QALY) can be estimated empirically and modelled [38, 39, 50].

The pure preparedness values require expansion of existing approaches but can be grounded in background economic theory and expressed in terms of social preferences for security. Together with the other evaluations, these preferences will then guide what costs (and opportunity costs) may be balanced by different options, e.g., to buy effective antibiotics, to invest in antibiotic innovation schemes or improved stewardship measures, or to pay for enhanced production and distribution security, better waste management, or development schemes to improve antibiotic access, or reduce the need of antibiotics, in LMI settings. The formation of preferences for the pure preparedness values may offer some challenges, as these actualize normative rather than factual uncertainties and related political disagreements. However, these are in principle no more difficult than the normative uncertainties and disagreements already prompted by societal decisions on how much to pay per QALY related to different health investments required by existing standard approaches.

A further practicality aspect is that the 3D Value Framework is sensitive to real variations between different antibiotics and different situations and surrounding contexts. This is important both to make the framework useful at different levels of decision-making (from specific investments to overall policy) and to guide adequate fact-finding underlying the evaluation. To achieve this, the framework distinguishes between different types of consumption and preparedness values on the patient and population levels, and it does so granularly enough to allow for relevant value-differences to be detected by the model. A positive side-effect of this granularity is that it also offers a way to divide the evaluation work into separate work-packages, which may then be synthesized to produce an outcome. Simply put, the framework may be used as a practical checklist in ways to be demonstrated below.

At the same time, increased granularity merely to boost theoretical sophistication is, in this context, undesirable as it would risk undermining practicality. In many real decision-making situations, it may suffice to operate with broad estimates, and a discrete set of positive weights or “value bonuses”, which are added to the values of an investment when more remote types of patients, population or pure preparedness values are identified. This is the solution that, for instance, the Swedish Dental and Pharmaceutical Benefits Agency (TLV) has implemented for considerations going beyond core health economical evaluation, such as extra willingness to pay for treatments of very severe or rare conditions [53].

A final practicality aspect regards how much quantitative automation should be built into the use of the 3D Value Framework in actual decision-making processes. This challenge is endemic to all health policy resource allocation where multiple values are considered, and subject of much theoretical debate. There are multiple alternative ways to aggregate and balance the values against costs to arrive at a final decision, and this holds even if all the social preferences discussed earlier have been clearly set [54]. Recently, Herlitz has critically analysed the idea of finding one unique algorithm for deriving practical conclusions from multiple value estimates, most clearly embraced in actual health policy making by NICE in the United Kingdom. However, the broader the health policy issues we consider, the more controversial each such notion will be, and a wider room for reasonable disagreement needs to be acknowledged. For that reason, Herlitz recommends a two-step approach, separating the assessment of value (and costs) from the process of determining how these should be balanced to produce an actual decision within a set of constraining procedural requirements regarding fairness, representation, transparency, rule of law, and so on [55].

We believe that this latter approach of using the 3D Value Framework in real decision making is more suitable for public policy. This is partly for the reasons given above, but also because an automated variant will tend to hide crucial ethics and policy stances on the proper aggregation and distribution of health-related values and how these are balanced against costs and uncertainties behind formalisms incomprehensible to most people. Such lack of transparency about controversial issues is not only a downside by itself but also creates a risk that the resulting decisions will not enjoy the broad legitimacy needed for social sustainability.

For this reason, the main usefulness of the 3D Value Framework lies in its capacity to function as a basis for a guiding checklist. The broken-down categories of value displayed in Fig. 2 can be systematically worked through, aided by (the collection of) relevant evidence. For each category, the question is asked: how much of this type of value would be generated by the suggested intervention or policy under consideration? For instance, at the time of writing, Sweden is pondering a system of public subsidy to help healthcare operators pay extra for continued production, supply and storage guarantees of selected antibiotics [56]. It is then crucial to determine how different levels and institutional constructions of this support mechanism, as well as the selection of exactly what is to be supported, would generate different amounts of different types of value, and what these are worth, in order to balance the costs and avoid that resources are wasted or that more valuable actions in other areas are foregone. More specifically, the use of the 3D Value Framework would proceed by, for a given policy alternative, systematically asking and answering the following questions:

1. How much primary patient value would be generated by the policy?
2. How much secondary patient value would be generated?
3. How much tertiary patient value would be generated?
4. How much system level population value would be generated?
5. How much structural population value would be generated?
6. How much societal population value would be generated?
7. For each of the questions 1–6, how much extra value of security would be generated?

Once one policy alternative (e.g. one specific level and mechanism for public subsidy) has been considered, other alternatives (e.g. other levels and mechanism) can be analysed in the same fashion to enable systematic comparison between available options.

Suppose, instead, that the suggested intervention is to support enhanced ABR-related waste management by paying extra (via subsidy or increased prices) to waste managing operators, healthcare institutions, farms and antibiotic suppliers to promote more sustainable practices without further undermining the already fragile antibiotic supply security. Or suppose that we ponder a global health policy to better control infection risk in LMI-settings to reduce the need of antibiotics, or to support such countries financially and capacity-wise so that their health systems can afford appropriate antibiotics while at the same time implementing effective antibiotic stewardship. Once again, the checklist use of the 3D Value Framework would proceed by asking and answering questions 1–7 above related to each available policy alternative. The answers would supply input for the denominator of a cost-effectiveness analysis, on the basis of which different policy options can be compared before determining whether the costs of any option is justified by the various values involved and which option is most cost-effective.

This basic use can also support decision making by guiding the very design of policy alternatives. For instance, many countries have set up or are pondering a system for rewarding successful antibiotic innovation [57], where one of the crucial issues if how to reward what types of successes. The checklist suggested above can here be employed to guide what types and levels of rewards would be appropriate, matched against how much value is added in the different dimensions.

## Discussion

We have argued for several improvements in the way policy makers value antibiotics – or, more precisely, sustainable access to effective antibiotics. These improvements are brought together in the 3D Value Framework and apply to decisions about antibiotic innovation, antibiotic stewardship and other actions to manage resistance, more secure antibiotic supply systems, and increased access to antibiotics in LMI settings. This framework partly builds on already initiated expansions of orthodox health economic approaches, but requires going beyond these, which we have argued is compatible with background economic theory, as well as established frameworks of healthcare, public health and health policy ethics. While this prompts some needs for further implementation work in specific policy contexts, we have argued that the 3D Value Framework is clear, comprehensive and practical enough to inform policy making as it is and that it can be used as a checklist to work through when evaluating the benefits of a potential investment and weighing these against opportunity costs. In this regard, we have recommended a transparent approach, where the evaluation of benefits and costs is separated from the question of how these are to be balanced, to promote legitimacy and fair procedure.

The decision- and policy making referred to in this article is primarily that of a public or semi-public (such as a non-profit healthcare charity or foundation) actor. However, we believe that the 3D Value Framework holds some promise to be useful also for commercial actors, such as pharmaceutical companies that sell antibiotics. First, companies with broad portfolios of drugs targeting many different conditions have business interests that relate to the strategic patient, population and pure preparedness values. This is because the value of many of these other drugs (e.g. sedatives for surgery, immunity suppressors or cancer drugs) are dependent on a sustainable access to effective antibiotics which enable the treatments where the other drugs are used. Second, to the extent that public health system actors use the considerations of the 3D Value Framework to evaluate investments, companies that sell antibiotics will have *instrumental* reasons to mind about sustainability, access and antibiotic effectiveness, since such aspects will increase the willingness to pay of their customers. Seen from the other end, this means that use of the 3D Value Framework will create financial incentives for commercial actors to care about sustainable access to effective antibiotics. At the same time, public payers can use the framework as a tool for resisting exaggerated cost increases driven by such considerations. Working out the details of the commercial use of the framework is beyond the scope of the present article.

A final observation is that, due to its generic theoretical design, the basic structure of the 3D Value Framework also holds some promise to be useful in other areas of policy that deal with resources of particular importance from a strategic and security standpoint. However, each such development would need context-specific work to create the desirable granularity in terms of adequate types of values. In addition, outside of health policy, values limited explicitly to health may not be adequate, and categories such as patient and population health would need to be revised. Obviously, such work resides far beyond the present article.

## Data Availability

No datasets were generated or analysed during the current study.
